# Supplementary material for: Validity and reliability of the Balance Error Score System (BESS) Thai version in patients with chronic non-specific neck pain
Source: PLoS One. 2024 Mar 28;19(3):e0301386. doi: 10.1371/journal.pone.0301386 (PMC10977775; doi:10.1371/journal.pone.0301386)
Supplement: S1 Text — (PDF) [file pone.0301386.s002.pdf]

การทดสอบการทรงตัว Balance Error Score System (BESS)

วิธีการทดสอบ

การทดสอบ BESS จะทำการทดสอบบนพื้นแข็งและพื้นโฟมอย่างละ 3 ท่า คือ ยืนด้วยขาสองข้าง ยืนด้วยขาข้างเดียวและยืนต่อเท้า รวมทั้งสิ้น 6 ท่าการทดสอบ โดยเมื่อผู้ถูกทดสอบอยู่ในท่าเริ่มต้นแล้ว ให้วางมือทั้งสองข้างไว้บริเวณขอบบนของกระดูกเชิงกราน จากนั้นจะเริ่มทำการทดสอบเมื่อผู้ถูกทดสอบหลับตาทั้งสองข้างแล้ว ต่อจากนั้นผู้วัดทำการนับข้อผิดพลาดในแต่ละท่าการทดสอบเป็นเวลา 20 วินาที แต่ละความผิดพลาดที่ผู้ถูกทดสอบทำให้นับเป็น 1 คะแนนต่อการเกิดขึ้น 1 ครั้ง ข้อผิดพลาดทั้งหมดจะถูกนำมารวมกันเป็นค่าคะแนน BESS

**หมายเหตุ ขาข้างที่ถนัด หมายถึง ขาข้างที่ใช้เตะลูกบอล

รูปแบบของข้อผิดพลาด

1. มือทั้งสองข้างหลุดจากขอบบนของกระดูกเชิงกราน
2. ลืมตาทั้งสองข้าง
3. ก้าวเท้า สะดุด หรือล้ม
4. เคลื่อนออกจากตำแหน่งทดสอบนานกว่า 5 วินาที
5. งอหรือกางข้อสะโพกมากกว่า 30 องศา
6. กระดกปลายเท้าหรือส้นเท้า

ตารางการบันทึก Balance error score system

ขาข้างที่ถนัด: ☐ ซ้าย ☐ ขวา **เพศ:** ☐ ชาย ☐ หญิง

**ท่ายืน-ข้อผิดพลาด/พื้นผิว**	**ค่าคะแนน**	
	**พื้นแข็ง (ครั้ง)**	**พื้นโฟม (ครั้ง)**
ยืนด้วยขา 2 ข้าง (เท้าทั้งสองชิดกันโดยให้ด้านในของเท้าอยู่กึ่งกลางของพื้นที่ทดสอบ)		
1. มือทั้งสองข้างหลุดจากขอบบนของกระดูกเชิงกราน		
2. ลืมตาทั้งสองข้าง		
3. ก้าวเท้า สะดุด หรือล้ม		
4. เคลื่อนออกจากตำแหน่งทดสอบนานกว่า 5 วินาที		
5. งอหรือกางข้อสะโพกมากกว่า 30 องศา		
6. กระดกปลายเท้าหรือส้นเท้า		
ยืนด้วยขาข้างเดียว (ยืนด้วยขาข้างที่ไม่ถนัดอยู่กึ่งกลางของพื้นที่ทดสอบ)		
1. มือทั้งสองข้างหลุดจากขอบบนของกระดูกเชิงกราน		
2. ลืมตาทั้งสองข้าง		
3. ก้าวเท้า สะดุด หรือล้ม		
4. เคลื่อนออกจากตำแหน่งทดสอบนานกว่า 5 วินาที		
5. งอหรือกางข้อสะโพกมากกว่า 30 องศา		
6. กระดกปลายเท้าหรือส้นเท้า		
ยืนต่อเท้า (ยืนในแนวทแยงบนพื้นที่ทดสอบ เท้าอยู่ในแนวเดียวกัน โดยให้เท้าข้างที่ถนัดอยู่หน้า)		
1. มือทั้งสองข้างหลุดจากขอบบนของกระดูกเชิงกราน		
2. ลืมตาทั้งสองข้าง		
3. ก้าวเท้า สะดุด หรือล้ม		
4. เคลื่อนออกจากตำแหน่งทดสอบนานกว่า 5 วินาที		
5. งอหรือกางข้อสะโพกมากกว่า 30 องศา		
6. กระดกปลายเท้าหรือส้นเท้า		
คะแนนรวมแต่ละพื้นผิว		
**คะแนนรวม BESS (พื้นแข็ง+พื้นโฟม)**		
