# Supplementary material for: Validity and reliability of the Balance Error Score System (BESS) Thai version in patients with chronic non-specific neck pain
Source: PLoS One. 2024 Mar 28;19(3):e0301386. doi: 10.1371/journal.pone.0301386 (PMC10977775; doi:10.1371/journal.pone.0301386)
Supplement: Attachment — Submitted filename: Response to Reviewers.pdf [file pone.0301386.s003.pdf]

Response to Reviewers

When submitting your revision, we need you to address these additional requirements.

1. Please ensure that your manuscript meets PLOS ONE's style requirements, including those for file naming. The PLOS ONE style templates can be found at https://journals.plos.org/plosone/s/file?id=wjVg/PLOSOne_formatting_sample_main_body.pdf and https://journals.plos.org/plosone/s/file?id=ba62/PLOSOne_formatting_sample_title_authors_affiliations.pdf

Response: Thank you for providing this link, we have read and edited our manuscript to match the PLOS One format requirements.

2. Thank you for stating the following financial disclosure: "Research Fund for Supporting Lecturer to Admit High Potential Student to Study and Research on His Expert Program Year 2021".

Please state what role the funders took in the study. If the funders had no role, please state: "The funders had no role in study design, data collection and analysis, decision to publish, or preparation of the manuscript."

If this statement is not correct you must amend it as needed.

Please include this amended Role of Funder statement in your cover letter; we will change the online submission form on your behalf.

Response: Thank you for your response and suggestion. We amended this sentence in the Cover Letter file as follows.

“This study received Research Funding for Supporting Lecturer to Admit High Potential Student to Study and Research on His Expert Program Year 2021 at Khon Kaen University. The funders had no role in study design, data analysis, decision to publish, or preparation of the manuscript.”

Section: Cover letter

3. In the online submission form, you indicated that "Data cannot be shared publicly because the authors want to conceal participants' information. However, if there are requirements for our data for academic proposals, they are available from the request at the corresponding email."

All PLOS journals now require all data underlying the findings described in their manuscript to be freely available to other researchers, either 1. In a public repository, 2. Within the manuscript itself, or 3. Uploaded as supplementary information.
This policy applies to all data except where public deposition would breach compliance with the protocol approved by your research ethics board. If your data cannot be made publicly available for ethical or legal reasons (e.g., public availability would compromise patient privacy), please explain your reasons on resubmission and your exemption request will be escalated for approval.

Response: Firstly, the authors apologize for our misunderstanding of your guideline and replying "No data sharing." Forthwith, the authors are freely available to share the supporting file and have added the heading "Supporting information" in the manuscript.

Section: Supporting information; Page 22; Line 460-464

4. Please review your reference list to ensure that it is complete and correct. If you have cited papers that have been retracted, please include the rationale for doing so in the manuscript text, or remove these references and replace them with relevant current references. Any changes to the reference list should be mentioned in the rebuttal letter that accompanies your revised manuscript. If you need to cite a retracted article, indicate the article's retracted status in the References list and also include a citation and full reference for the retraction notice.

Response: Thank you for reminding us of this point. We have carefully rechecked our reference lists and edited some references for the subsequence item to fit with the "Vancouver" style as detailed in the Reference style part of the PLOS ONE submission guideline. There was one

reference that had been redacted and needed to be replaced. That is citation number 76 written as “Khasnis A, Gokula RM. Romberg’s test. J Postgrad Med. 2003;49: 169. Available: https://www.jpgmonline.com/article.asp?issn=0022-3859;year=2003;volume=49;issue=2;spage=169;epage=72;aulast=Khasnis.” After carefully rechecking of this link, the link is not further available, thus we replaced with citation number 77 written as “Murray N, Salvatore A, Powell D, Reed-Jones R. Reliability and validity evidence of multiple balance assessments in athletes with a concussion. J Athl Train. 2014;49: 540–549. doi:10.4085/1062-6050-49.3.32.” instead of citation number 76. Moreover, the paper, that may make you wonder about this, is citation number 13 previously written as “Choochouy N, Saita S, Sirithian D. Prevalence of and factors associated with occupational health problems among hill farmers in Thailand. 2022”. Since rechecking we have edited it to correctly read as “Choochouy N, Saita S, Sirithian D. Prevalence of and factors associated with occupational health problems among hill farmers in Thailand. Southeast Asian J Trop Med Public Health. 2022;53: 368–386.” The Southeast Asian Journal of Tropical Medicine and Public Health is in SCOPUS in Q4 as shown in the following link https://www.scimagojr.com/journalsearch.php?q=23093&tip=sid&clean=0.

Section: Methods, Page: 12, Line 280

Reference, Page: 23, Line: 508-510

Reviewers' comments:

Reviewer's Responses to Questions

Comments to the Author

1. Is the manuscript technically sound, and do the data support the conclusions?

The manuscript must describe a technically sound piece of scientific research with data that supports the conclusions. Experiments must have been conducted rigorously, with appropriate controls, replication, and sample sizes. The conclusions must be drawn appropriately based on the data presented.

Reviewer #1: Yes

Reviewer #2: Partly

Response: Thank you, we have aimed to clarify. This study began with the cross-cultural adaptation of the BESS instrument from the original English to the Thai language, followed by testing its content and convergent validity, and finally assessing its reliability.

This study was conducted rigorously. Prior to commencing the study, we reviewed the psychometric properties that the BESS instrument should meet. Additionally, we reviewed previous research and referenced processes most suitable for our study (Chatprem et al., 2020; Georgieva-Zhostova et al., 2014; Jaikaew & Satiansukpong, 2019; Kurre et al., 2009; Meeapirak et al., 2021; Tongprasert et al., 2014), providing detailed descriptions with some pictures for clarity to readers and for replication in future research.

From statisticians, we obtained confirmation regarding the study sample size, with supporting references. We made efforts to control all potential confounding factors and biases and have rewritten the conclusion more appropriately based on the data support as outlined in the manuscript's relevant sections.

References

1. Kurre A, Van Gool CJAW, Bastiaenen CHG, Gloor-Juzi T, Straumann D, De Bruin ED. Translation, cross-cultural adaptation and reliability of the german version of the dizziness handicap inventory. Otol Neurotol. 2009;30: 359–367. doi:10.1097/MAO.0B013E3181977E09

2. Tongprasert S, Rapipong J, Buntragulpoontawee M. The cross-cultural adaptation of the DASH questionnaire in Thai (DASH-TH). Journal of Hand Therapy. 2014;27: 49–54. doi:10.1016/J.JHT.2013.08.020

3. Georgieva-Zhostova S, Kolev OI, Stambolieva K. Translation, cross-cultural adaptation and validation of the Bulgarian version of the Dizziness Handicap Inventory. Qual Life Res. 2014;23: 2103–2107. doi:10.1007/S11136-014-0660-5

4. Jaikaew R, Satiansukpong N. Movement assessment battery for children-second edition (MABC2): Cross-cultural validity, content validity, and interrater reliability in Thai children. Occup Ther Int. 2019;2019. doi:10.1155/2019/4086594

5. Meeapirak P, Hunsawong T, Chatchawan U, Siritaratiwat W, Boonprakob Y. Translation, cross-cultural adaptation, and psychometric properties of the Thai version of the Identification of

Functional Ankle Instability (IdFAI-THAI) questionnaire. Foot Ankle Surg. 2021 [cited 20 Apr 2022]. doi:10.1016/J.FAS.2021.10.007

6. Chatprem T, Puntumetakul R, Boucaut R, Wanpen S, Chatchawan U. A Screening Tool for Patients With Lumbar Instability: A Criteria-related Validity of Thai Version. Spine (Phila Pa 1976). 2020;45: E1431–E1438. doi:10.1097/BRS.0000000000003606

2. Has the statistical analysis been performed appropriately and rigorously?

Reviewer #1: Yes

Reviewer #2: I Don't Know

Response: Thank you for your review. We have strengthened our reporting of how we analyzed our data appropriately and rigorously as described follows.

For the cross-cultural translation and development of the BESS Thai version phase, the researchers followed and adapted the standard protocol that was reported in previous research (Beaton et al., 2000; Sousa & Rojjanasrirat, 2011) to describe the detail and sequence of the translation phase.

To assess content validity, researchers can use one of the following statistical tests: the Index of Item Objective Congruence (IOC), the Content Validation Index (CVI), or the Content Validity Ratio (CVR). IOC can be used to calculate either multidimensional or unidimensional indexes (Turner & Carlson, 2003). In the current study, the investigators decided to use the IOC because it aims to determine the utility of the BESS-TH in measuring balance among participants with chronic non-specific neck pain, as rated by experts in the relevant context.

Assessing convergent validity, involves determining the degree of correlation between the newly developed scale and other variables, as well as other measures assessing the same construct (Krabbe, 2017). The current study investigators decided to use convergent validity because other tools used to measure static balance in participants with neck pain include: force plates (Jørgensen et al., 2011; Palmgren et al., 2009; Poole et al., 2008; Sremakaew et al., 2021), Single Leg Balance Test (SLBT) (Duray et al., 2018), Romberg test (Jørgensen et al., 2011), Tandem stance test (Treleaven, Jull, et al., 2005; Treleaven, Murison, et al., 2005), and balance error scoring system (BESS) (Wah et al., 2021).

When comparing the BESS with measures using a force plate (considered the gold standard), it is called "criterion-related validity" as this has already been conducted in previous research and reported (Kleffelgaard et al., 2018). In the current study, we included the clinical field tests of balance measurement in participants with neck pain including the: Single Leg Balance Test (SLBT), Romberg test, Tandem stance test, and balance error scoring system (BESS).

For assessing reliability in this study, the investigators measured inter and intra-rater reliability. Inter-rater and intra-rater reliability were calculated by the Interclass Correlation Coefficient (ICC). ICC model 2, 1 was used for inter-rater reliability, and model 3, 1 was used for intra-rater reliability.

However, we have considered the reviewer's comment, and have added the ceiling and flooring effect, standard error of measurement (SEM), and minimum detectable change (MDC) in our manuscript to cover the objectives of our study.

Section: Abstract, Page: 2, Line: 44-45;
Methods, Page: 10, Line: 238-242 and Page: 13, Line: 300-301;
Results, Page: 17-18, Line: 361-369 and table 6;
Discussion, Page: 19, Line: 380-381, Page: 20, table 7, Page 21, Line: 427-430

References

1. Sousa VD, Rojjanasrirat W. Translation, adaptation and validation of instruments or scales for use in cross-cultural health care research: A clear and user-friendly guideline. J Eval Clin Pract. 2011;17: 268–274. doi:10.1111/j.1365-2753.2010.01434.x

2. Beaton DE, Bombardier C, Guillemin F, Ferraz MB. Guidelines for the process of cross-cultural adaptation of self-report measures. Spine (Phila Pa 1976). 2000;25: 3186–3191. doi:10.1097/00007632-200012150-00014

3. Turner RC, Carlson L. Indexes of Item-Objective Congruence for Multidimensional Items. Int J Test. 2003;3: 163–171. doi:10.1207/s15327574ijt0302_5

4. Krabbe PFM. Validity. The Measurement of Health and Health Status. 2017; 113–134. doi:10.1016/B978-0-12-801504-9.00007-6

5. Poole E, Treleaven J, Jull G. The influence of neck pain on balance and gait parameters in community-dwelling elders. Man Ther. 2008;13: 317–324. doi:10.1016/j.math.2007.02.002

6. Jørgensen MB, Skotte JH, Holtermann A, Sjøgaard G, Petersen NC, Søgaard K. Neck pain and postural balance among workers with high postural demands - A cross-sectional study. BMC Musculoskelet Disord. 2011;12. doi:10.1186/1471-2474-12-176

7. Sremakaew M, Treleaven J, Jull G, Vongvaivanichakul P, Uthaikhup S. Altered neuromuscular activity and postural stability during standing balance tasks in persons with non-specific neck pain. Journal of Electromyography and Kinesiology. 2021;61: 102608. doi:10.1016/j.jelekin.2021.102608

8. Palmgren PJ, Andreasson D, Eriksson M, Hägglund A. Cervicocephalic kinesthetic sensibility and postural balance in patients with nontraumatic chronic neck pain - A pilot study. Chiropr Osteopat. 2009;17: 1–10. doi:10.1186/1746-1340-17-6

9. Duray M, Simşek S, Altuğ F, Cavlak U. Effect of proprioceptive training on balance in patients with chronic neck pain. Agri. 2018;30: 130–137. doi:10.5505/agri.2018.61214

10. Treleaven J, Murison R, Jull G, LowChoy N, Brauer S. Is the method of signal analysis and test selection important for measuring standing balance in subjects with persistent whiplash? Gait Posture. 2005;21: 395–402. doi:10.1016/J.GAITPOST.2004.04.008

11. Treleaven J, Jull G, LowChoy N. Standing balance in persistent whiplash: A comparison between subjects with and without dizziness. J Rehabil Med. 2005;37: 224–229. doi:10.1080/16501970510027989

12. Wah SW, Puntumetakul R, Boucaut R. Effects of proprioceptive and craniocervical flexor training on static balance in university student smartphone users with balance impairment: A randomized controlled trial. J Pain Res. 2021;14: 1935–1947. doi:10.2147/JPR.S312202

13. Kleffelgaard I, Langhammer B, Sandhaug M, Pripp AH, Søberg HL. Measurement properties of the modified and total Balance ERror SCoring System–the BESS, in a healthy adult sample. Eur J Physiother. 2018;20: 25–31. doi:10.1080/21679169.2017.1352020

3. Have the authors made all data underlying the findings in their manuscript fully available?

The PLOS Data policy requires authors to make all data underlying the findings described in their manuscript fully available without restriction, with rare exceptions (please refer to the Data Availability Statement in the manuscript PDF file). The data should be provided as part of the

manuscript or its supporting information or deposited in a public repository. For example, in addition to summary statistics, the data points behind means, medians, and variance measures should be available. If there are restrictions on publicly sharing data—e.g. participant privacy or use of data from a third party—those must be specified.

Reviewer #1: Yes

Reviewer #2: Yes

Response: Thank you. The authors plan to add the raw data and the BESS Thai version in the supporting information section as follows.

“Supporting information.

S1 Data. Analysis Data.
(XLSX)
S2 Text. A Thai version of the Balance Error Scoring System.
(PDF)”

Section: Supporting information; Page 22; Line 460-464

4. Is the manuscript presented in an intelligible fashion and written in standard English?

PLOS ONE does not copyedit accepted manuscripts, so the language in submitted articles must be clear, correct, and unambiguous. Any typographical or grammatical errors should be corrected at revision, so please note any specific errors here.

Reviewer #1: Yes

Reviewer #2: Yes

Response: Thank you.

5. Review Comments to the Author

Please use the space provided to explain your answers to the questions above. You may also include additional comments for the author, including concerns about dual publication, research ethics, or publication ethics. (Please upload your review as an attachment if it exceeds 20,000 characters)

Reviewer #1: Thank you for the opportunity to review this manuscript, "Validity and reliability of the Balance Error Score System (BESS) Thai version in patients with chronic non-specific neck pain". The manuscript was well written and is appropriate for publication in PLOS ONE Journal.

Response: Thank you for your review and your appreciation of this manuscript.

Reviewer #2: Dear Authors
Thanks for your interesting article. It is very useful in clinical work as well as research.
I have some suggestions as follows:
1. How did you diagnose the patients to have non-specific neck pain? I mean how did you assure that these patients did not have disc herniation or so on?

Response 1: Thank you. Non-specific neck pain (NSNP) is defined as a type of neck pain without a detectable etiology and with no features of red flag conditions such as: malignancy, infection, inflammation, myelopathy, other histories of orthopedics conditions and drop attacks during head movement, or symptoms following whiplash (Binder, 2007; Borghouts et al., 1998). We have added further information in the Introduction. Even though we did not include the patients who have red flag signs, there may be other conditions. For an accurate diagnosis, clinical images such as X-ray, CT, or MRI confirm the true NSNP patients (Parikh et al., 2019). The current study did not provide the opportunity for such diagnostic tests (which are expensive and may be unnecessary). Thus, we put this in the Limitation of the study as follows.

“NSNP is defined as a type of NP without a detectable etiology and with no features of red flag conditions such as malignancy, infection, inflammation, myelopathy, other histories of orthopedics conditions and drop attacks during head movement, or symptoms following whiplash”

“Patients with CNSNP who lacked an identifiable cause and did not exhibit any symptoms of serious underlying illnesses.”

“This study included participants with chronic non-specific neck pain (CNSNP) who met the criteria of neck pain without an identifiable cause and had no indications of serious medical conditions such as cancer, infection, inflammation, myelopathy, previous orthopedic conditions, drop attack during head movement, or symptoms following whiplash injury. Our focus was solely on medical histories, meaning there may have an opportunity for a person with mild cervical disc herniation to have been included in the study. In future studies, investigators may consider the possibility of employing imaging techniques to rule out disc herniation.”

Section: Introduction, Page 3, Line: 58-61

Methods, Page: 6, Line: 144-145

Discussion, Page 21; Line 436-443

References

1. Borghouts JAJ, Koes BW, Bouter LM. The clinical course and prognostic factors of non-specific neck pain: a systematic review. Pain. 1998;77: 1–13. doi:10.1016/S0304-3959(98)00058-X
2. Binder A. The diagnosis and treatment of nonspecific neck pain and whiplash. Eura Medicophys. 2007;43: 79–89.
3. Parikh P, Santaguida P, Macdermid J, Gross A, Eshtiaghi A. Comparison of CPG’s for the diagnosis, prognosis and management of non-specific neck pain: a systematic review. BMC Musculoskelet Disord. 2019;20: 81. doi:10.1186/s12891-019-2441-3

2. Please mention about the “Floor or ceiling effects” in the article. It should be calculated.

Response 2: Thank you. Although neither the “Floor nor ceiling effects” are one of our objectives, they reflect possible reasons to support the correlation between the tests in the convergent validity process. Therefore, we have added the floor and ceiling effects in the Discussion Section and Table 7 as follows.

“Table 7 Ceiling and floor effect statistics for Each Balance Measurement”

**Measurements**	**Floor effect (%)**	**Ceiling effect (%)**
BESS-TH (scores)	0	0
SLBT with eyes open (seconds)	0	**22.3**
SLBT with eyes closed (seconds)	0	0.8
Romberg with eyes open (seconds)	0	**92.3**
Romberg with eyes closed (seconds)	0	**60.8**
Tandem with eyes open (seconds)	0	**64.6**
Tandem with eyes closed (seconds)	0	**26.2**

Section: Discussion, Page 20, Table 7

3. In addition to ICC, which has reported in the manuscript, please add the Cronbach’s alpha as well as minimally metrically detectable change (MMDC) for reliability and internal consistency.

Response 3: Thank you for your suggestion. Regarding Cronbach’s alpha, as Reviewer 2 mentioned, it is mostly employed to evaluate the internal consistency of a questionnaire or survey comprising many sub-scales and items (Tavakol & Dennick, 2011). However, the BESS-TH is an instrument that assesses static balance ability, which may differ from other questionnaires such as RMDQ and SF-36, etc. The BESS-TH test has 6 subtests, each measuring different positions and supporting surfaces. Therefore, we have decided not to include Cronbach’s alpha as one of our outcomes.

Regarding the minimally detectable change (MDC) that Reviewer 2 mentioned, the concept of minimal detectable change (MDC) has been used to define the amount of change in a variable that must be achieved before we can be confident that error does not account for the entire measured difference, and that some true change must have occurred (Portney, 2020). In this part, we have decided to add the measurement of MDC and standard error of measurement (SEM) in the reliability phase. Thus, we added the details in the following section.

Section: Abstract: Page 2; Line 44-45

Methods: Page: 10, Line: 238-242 and Page: 13, Line: 300-301

Results: Page: 17-18, Line: 361-369 and Table 6

Discussion: Page: 19, Line: 380-381 and Page: 21, Line: 427-430

References

1. Tavakol M, Dennick R. Making sense of Cronbach's alpha. International journal of medical education. 2011. pp. 53–55. doi:10.5116/ijme.4dfb.8dfd

2. Portney LG. Foundations of clinical research : applications to evidence-based practice. 4th Edition. Philadelphia, PA: F. A. Davis Company; 2020.

4. Please check the spell of the word "Kolmogorov–Smirnov test".

Response 4: Kolmogorov–Smirnov is the correct wording for the test – we have checked this in the manuscript.

Section: Methods: Page: 13, Line: 297

5. As you mentioned, you have not checked the criterion validity or using a gold standard to check the score. So, it seems that this article is somehow a cross-cultural adaptation. So, why did not mention in the topic?

Response 5: Thank you for your question. The current study aimed to translate the BESS balance test into the Thai language and perform content and convergent validity, and reliability. In the title, we used the phrase "the Balance Error Score System (BESS) Thai version", indicating the necessity of a cross-cultural adaptation process to obtain this Thai version. Additionally, in the topic, the research employed the term "validity" and provided detailed descriptions of measuring content and convergent validity.

Regarding comparison with force plate measures (considered the gold standard), this was studied in male athletes with correlations ranging from low to high (r=0.31-0.79) (Riemann et al., 1999). This already establishes criterion-related validity, and the current study also compares the clinical field tests that did not include the force plate. Thus, for further research, the

researchers mentioned another validity type that was not performed at this time, which is "criterion-related validity" in participants with chronic non-specific neck pain.

Reference

1. Riemann BL, Guskiewicz KM, Shields EW. Relationship between clinical and forceplate measures of postural stability. J Sport Rehabil. 1999;8: 71–82. doi:10.1123/jsr.8.2.71

6. What about the "Dimensionality"? Have you checked it?

Response 6: Thank you for your question.

"Unidimensionality" was explained in the book of Portney (2020) as the one the way to examine the internal construct validity as follows.

"The Rasch model has provided a way to examine the internal construct validity of a measure, including ordering of categories, unidimensionality, and whether items are biased across subgroups (Portney, 2020)."

The dimensionality of a tool is assessed using factor analysis. Factor analysis aims to identify common patterns among a group of variables by examining their shared variance. The primary goal of this analysis is to determine the most straightforward and concise method for interpreting and displaying the collected data (Manzar et al., 2018). From the review, the studies that perform dimensionality are mainly in questionnaires (Manzar et al., 2018; Rawang et al., 2020; Terwee et al., 2007; Vet et al., 2005). The current study consists of 6 subtests of BESS-TH and 3 other balance tests. Thus, we have considered not analyzing dimensionality.

References

1. Portney LG. Foundations of clinical research : applications to evidence-based practice. 4th Edition. Philadelphia, PA: F. A. Davis Company; 2020.

2. Manzar MD, BaHammam AS, Hameed UA, Spence DW, Pandi-Perumal SR, Moscovitch A, et al. Dimensionality of the Pittsburgh Sleep Quality Index: a systematic review. Health Qual Life Outcomes. 2018;16. doi:10.1186/S12955-018-0915-X

3. Vet HCW de, Adèr HJ, Terwee CB, Pouwer F. Are factor analytical techniques used appropriately in the validation of health status questionnaires? A systematic review on the

quality of factor analysis of the SF-36. Quality of Life Research. 2005;14: 1203–1218. doi:10.1007/s11136-004-5742-3

4. Rawang P, Janwantanakul P, Correia H, Jensen MP, Kanlayanaphotporn R. Cross-cultural adaptation, reliability, and construct validity of the Thai version of the Patient-Reported Outcomes Measurement Information System-29 in individuals with chronic low back pain. Quality of Life Research. 2020;29: 793–803. doi:10.1007/s11136-019-02363-x

5. Terwee CB, Bot SDM, de Boer MR, van der Windt DAWM, Knol DL, Dekker J, et al. Quality criteria were proposed for measurement properties of health status questionnaires. J Clin Epidemiol. 2007;60: 34–42. doi:10.1016/j.jclinepi.2006.03.012

[NOTE: If reviewer comments were submitted as an attachment file, they will be attached to this email and accessible via the submission site. Please log into your account, locate the manuscript record, and check for the action link "View Attachments". If this link does not appear, there are no attachment files.]

While revising your submission, please upload your figure files to the Preflight Analysis and Conversion Engine (PACE) digital diagnostic tool, https://pacev2.apexcovantage.com/. PACE helps ensure that figures meet PLOS requirements. To use PACE, you must first register as a user. Registration is free. Then, login and navigate to the UPLOAD tab, where you will find detailed instructions on how to use the tool. If you encounter any issues or have any questions when using PACE, please email PLOS at figures@plos.org. Please note that Supporting Information files do not need this step.
